# Whole-genome sequencing-based epidemiological analysis of anti-tuberculosis drug resistance genes in Japan in 2007: Application of the Genome Research for Asian Tuberculosis (GReAT) database

**DOI:** 10.1038/s41598-019-49219-5

**Published:** 2019-09-06

**Authors:** Takemasa Takii, Kouhei Seki, Yasutaka Wakabayashi, Yuta Morishige, Tsuyoshi Sekizuka, Akifumi Yamashita, Kengo Kato, Kazuhiro Uchimura, Akihiro Ohkado, Naoto Keicho, Satoshi Mitarai, Makoto Kuroda, Seiya Kato

**Affiliations:** 1 Department of Mycobacteriology, Research Institute of Tuberculosis, Japan Anti-Tuberculosis Association, 3-1-24 Matsuyama, Kiyose, Tokyo, 204-8533 Japan; 2 Pathogen Genomics Center, National Institute of Infectious Diseases, 1-23-1 Toyama, Shinjuku-ku, Tokyo, 162-8640 Japan; 3 Department of Epidemiology and Clinical Research, Research Institute of Tuberculosis, Japan Anti-Tuberculosis Association, 3-1-24 Matsuyama, Kiyose, Tokyo, 204-8533 Japan; 4 Research Institute of Tuberculosis, Japan Anti-Tuberculosis Association, 3-1-24 Matsuyama, Kiyose, Tokyo, 204-8533 Japan

**Keywords:** Genetic markers, Bacterial genetics

## Abstract

We investigated the lineages of *Mycobacterium tuberculosis* (Mtb) isolates from the RYOKEN study in Japan in 2007 and the usefulness of genotypic drug susceptibility testing (DST) using the Genome Research for Asian Tuberculosis (GReAT) database. In total, 667 isolates were classified into lineage 1 (4.6%), lineage 2 (0.8%), lineage 2/Beijing (72.1%), lineage 3 (0.5%), and lineage 4 (22.0%). The nationality, gender, and age groups associated with the isolates assigned to lineage 1 were significantly different from those associated with other lineages. In particular, isolates of lineage 1.2.1 (EAI2) formed sub-clusters and included a 2,316-bp deletion in the genome. The proportion of the isolates resistant to at least one anti-tuberculosis (TB) drug was 10.8%, as determined by either the genotypic or phenotypic method of DST. However, the sensitivities to isoniazid, streptomycin, and ethambutol determined by the genotypic method were low. Thus, unidentified mutations in the genome responsible for drug resistance were explored, revealing previously unreported mutations in the *katG*, *gid*, and *embB* genes. This is the first nationwide report of whole-genome analysis of TB in Japan.

## Introduction

Tuberculosis (TB) has been difficult to eradicate, and according to the World Health Organization (WHO) global TB report, there were an estimated 10.4 million new TB infections and 1.3 million deaths worldwide in 2016 (http://www.who.int/tb/publications/global_report/en/). TB remains a substantial burden in various Asian countries, with annual incidence rates of 150–300 infections per 100,000 persons. In 2016, the rates were markedly high in Korea and the Philippines, with more than 500 cases per 100,000 persons, whereas the incidence rate in Japan was comparatively low at 13.9 cases per 100,000 persons (http://www.jata.or.jp/rit/ekigaku/en/annual-reports/). However, the emergence of TB in Japan among foreigners has become problematic because of the increasing numbers of travellers and workers from countries with high incidence rates of TB infection^1^. Furthermore, the number of patients with dual infections of TB and human immunodeficiency virus is also high, and multidrug-resistant and extensively drug-resistant forms of TB are emerging in Asian countries^2–6^.

Technological developments, such as whole-genome sequencing (WGS) and next-generation sequencing, have enabled the elucidation of the lineages and mutations of drug resistance (DR) genes based on the detection of insertions and deletions in the genome of *Mycobacterium tuberculosis* (Mtb)^7^. Furthermore, WGS is a useful method for tracing the origins of isolates and phylogenetic analysis of single-nucleotide variants (SNVs) as well as for epidemiological studies for the detection of recent transmissions and for tracing outbreaks of TB^8,9^. The Relational Sequencing TB Data Platform (ReSeqTB) (https://platform.reseqtb.org/), Pathgenseq (http://pathogenseq.lshtm.ac.uk), and Sprint-TB (http://www.sprinttb.org) databases have been developed to better understand the biology and behaviour of Mtb. In particular, the ReSeqTB database^10^ contains information on more than 6,000 isolates, including DR forms, from European, African, and Asian countries, mainly from the United Kingdom, Germany, South Africa, and the Russian Federation. Likewise, in 2016, our group created the Genome Research for Asian Tuberculosis (GReAT) database, which contains information on more than 3,000 clinical Mtb isolates from East and South-East Asian countries, including China, Taiwan, the Philippines, Vietnam, Korea, Mongolia, and Japan; this database is financially supported by the Japan Agency for Medical Research and Development. The purpose of the GReAT project is to collect genomic data for the control of TB worldwide and for the development of new technologies for the diagnosis and treatment of TB. The isolates included in the GReAT database were collected from surveys of TB and drug-resistant TB conducted in each of the participating Asian countries. In the present study, the characteristics of Mtb isolates included in the RYOKEN 2007 nationwide anti-TB drug susceptibility study in Japan and the GReAT database were investigated.

## Materials and Methods

### Strain collection and drug susceptibility testing

All Japanese strains were collected in 2007 by Tuberculosis Research Committee (RYOKEN) Japan a nationwide coalition of TB hospitals in Japan that report DR rates once every 5 years^11^. These isolates were collected from August 2007 to July 2008. In total, 329 isolates collected from patients aged >40 years were randomly selected from the registered patients in this study to investigate the proportion of foreign-born TB patients. In addition, 338 isolates collected from patients aged 0–39 years were also included for analysis. WGS of the isolates was performed using an all-in-one web-based tool for genotyping of Mtb, namely, Total Genotyping Solution for TB (TGS-TB). All isolates were subjected to phylogenetic analysis, *in silico* spoligotyping, and DR prediction^12^. The concordance, specificity, and sensitivity of prediction of drug susceptibility by TGS-TB were calculated by comparing the results of phenotypic drug susceptibility testing (DST), which was used as a gold standard. The phenotypic susceptibility of the isolates to anti-tuberculous drugs [i.e., isoniazid (INH), rifampicin (RFP), streptomycin (SM), ethambutol (EB), and levofloxacin (LVFX)] was calculated using the proportion method^13^. The genotyping results of the isolates were compared with the results of SNV-based genotype analysis on TGS-TB^12^.

### DNA preparation and WGS of the Mtb isolates

Genomic DNA was extracted using the DNA ISOPLANT Kit (Wako Pure Chemical Industries, Ltd., Osaka, Japan), purified with a QIAquick column (QIAGEN GmbH, Hilden, Germany), and quantified using a Qubit 2.0 fluorometer (Thermo Fisher Scientific, Waltham, MA, USA). Paired-end libraries were prepared from 50 ng of purified DNA with the QIAseq FX DNA Library Kit (QIAGEN GmbH, Hilden, Germany) in accordance with the manufacturer’s protocol. The average fragment size (500–600 bp) of the DNA libraries was estimated by 2% agarose gel electrophoresis. Then, the fragments were eluted using the Wizard SV Gel and PCR Clean-Up System (Promega Corporation, Madison, WI, USA). The 24 purified DNA libraries were pooled, and the DNA concentration was quantified with a Qubit 2.0 fluorometer. The pooled libraries (11 pM) were sequenced on an Illumina MiSeq system (Illumina, Inc., San Diego, CA, USA) with the MiSeq Reagent Kit ver. 3 (600 cycles), which showed that the first paired-end reads were 350 nt in length, whereas the second paired-end reads were 250 nt in length.

### Genomic analysis of the isolates with informatic tools

The reads obtained from sequencing were analysed using TGS-TB^12^, which is a pipeline for conventional epidemiological analysis. Prediction of genetic markers for antimicrobial resistance (e.g., *ahpC*, *embA*, *embB*, *embC*, *embR*, *ethA*, *ethR*, *gid*, *gyrA*, *gyrB*, *inhA*, *kasA*, *katG*, *pncA*, *rpoB*, *rpoC*, *rpsA*, *rpsL*, and *rrs*) listed in the TB profiler database^14^, lineage analysis (i.e., lineage 1, lineage 2, lineage 2/Beijing, lineage 3, and lineage 4) based on single-nucleotide polymorphisms followed by KvarQ^15^, and *in silico* spoligotyping were automatically performed based on the sequence data. Core-genome phylogenetic and linkage networks were also analysed using TGS-TB^12^. Prior to *in silico* genotyping, the adapter sequences were trimmed from the short reads, and low-quality bases with a Phred score of <15 were eliminated^12^ using the Skewer program to obtain sequences that were at least 50-mers^16^. The remaining reads were mapped using the BWA-mem program^17^ with the reference genome sequence of Mtb strain H_37_Rv (NC_000962.3)^18^. Reliable SNV sites with at least a 5× coverage depth and a Phred score of ≥20 were selected^12^. In this study average coverage depth was 85. Maximum likelihood phylogenetic analysis of all concatenated SNV alleles was performed using RAxML v8.2.0^19^ with 1,000 bootstrap iterations. To identify epidemiological linkages among the isolates, data from queries for isolate-specific genes or the abovementioned reference genomes were downloaded as a NEXUS format file to visualize linkage networks, such as by the median-joining method for network visualization using PopART (http://popart.otago.ac.nz). *In silico* spoligotyping was performed by a search using the Basic Local Alignment Search Tool with 43 spacer sequences^20^.

### Statistical analysis

Data are summarized as the mean, median, and/or range, as appropriate, and compared using Fisher’s exact test or the chi-square test. All tests were two-sided, and a probability (*p*) value of < 0.05 was considered statistically significant.

### Ethics approval

The study protocol was reviewed and approved by the institutional review boards of the ethics committee of the Japan Anti-Tuberculosis Association (no. 28–10). As researchers had no access to information to identify an individual patient or treatment, this committee waived the need for informed consent for this study.

## Results

### Lineages of isolates from male and female patients

As shown in Table 1, of the 667 Mtb isolates (371 collected from males and 216 from females), 31 (4.6%), 5 (0.8%), 481 (72.1%), 3 (0.5%), and 147 (22.0%) were identified as lineages 1, 2, 2/Beijing, 3, and 4, respectively. Notably, lineage 1 isolates were significantly more common in females than in males aged 20–39 years (*n* = 5 vs. 17, respectively; odds ratio = 0.17; 95% confidence interval = 0.06–0.47, *p* < 0.01; Table 1).

**Table 1:** Lineage distribution of Mtb isolates in the RYOKEN 2007 dataset of the GReAT database by age group and gender.

Age	Gender	Lineage 1		OR	Lineage 2		Lineage 2/Beijing			Lineage 3		Lineage 4		OR	Total	
			*p* ^*^					*p* ^*^	OR				*p* ^*^			
			(95% CI)					(95% CI)					(95% CI)			
0-19	Male	1	1.00	1.00	0		7	0.60	1.75	0		1	0.53	0.44	9	
	Female	1	(0.05–18.92)		0		6	(0.22–14.22)		0		2	(0.03–5.93)		9	
20-39	Male	5	<0.01	0.17	3		137	<0.01	1.31	3		45	0.92	1.09	193	
	Female	17	(0.06–0.47)		0		81	(0.82–2.11)		0		29	(0.64–1.86)		127	
40-	Male	5	0.84	1.18	1		127	0.43	1.27	0		35	0.31	0.73	168	
	Female	2	(0.22–6.22)		0		56	(0.70–2.32)		0		21	(0.39–1.36)		79	
**Age-UN**																
	Male	0			0		1			0		0			1	
	Female	0			0		1			0		0			1	
Gender-UN		0			1		65			0		14			80	
Total		31	4.6%		5	0.8%	481	72.1%		3	0.5%	147	22.0%		667	100%
	Male	11	35.5%		4	80.0%	272	56.5%		3	100.0%	81	55.1%		371	55.6%
	Female	20	64.5%		0	0.0%	144	29.9%		0	0.0%	52	35.4%		216	32.4%
Gender-UN		0	0.0%		1	20.0%	65	13.5%		0	0.0%	14	9.5%		80	12.0%
Total		31	100.0%		5	100.0%	481	100.0%		3	100.0%	147	100.0%		667	100.0%

*p-value was calculated with the chi-square test by comparing the distribution in the other lineages combined. OR; odds ratio, CI; confidence interval, UN; unknown. Percentages are the ratios of the isolates belonging to each lineage.

### Isolates from Japanese and foreign-born patients

As shown in Table 2, of the 667 isolates, 40 (6.0%) were from foreign-born patients, with 20 (50%), 12 (30%), and 8 (20%) belonging to lineages 1, 2/Beijing, and 4, respectively. Of these 40 isolates, 37 were from foreign-born patients aged 20–39 years, with 18 (45%) identified as lineage 1. Of the 31 isolates identified as lineage 1, 20 (64.5%) were from foreign-born patients, including nine patients from the Philippines, two each from Thailand, Nepal, and Vietnam, and five from other countries.

**Table 2:** Lineage distribution of Mtb isolates in the RYOKEN 2007 dataset of the GReAT database by age group and country of origin.

Age	Nationality	Lineage 1			Lineage 2		Lineage 2/Beijing			Lineage 3		Lineage 4			Total	
			*p* ^*^	OR				*p* ^*^	OR				*p* ^*^	OR		
			(95% CI)					(95% CI)					(95% CI)			
0–19	Japan	1	0.06	0.07	0		13	0.02		0		2	0.18	0.14	16	
	Foreign	1	(0.00–2.06)		3		0			0		1	(0.01–3.31)		2	
20–39	Japan	4	<0.01	0.02	0		207	<0.01	5.53	3		67	0.50	1.32	284	
	Foreign	18	(0.01–0.05)		1		12	(2.65–11.54)		0		7	(0.56–3.15)		37	
40-	Japan	6	<0.01		0		182	0.09		0		56	0.59		245	
	Foreign	1			0		0			0		0			1	
Age-UN	Japan	0			0		2			0		0			2	
	Foreign	0			0		0			0		0			0	
Nationality-UN		0			1		65			0		14			80	
Total		31	4.6%		5	0.8%	481	72.1%		3	0.5%	147	22.0%		667	100.0%
	Japan	11	35.5%		4	80.0%	404	84.0%		3	100.0%	125	85.0%		547	82.0%
	Foreign	20	64.5%		0	0.0%	12	2.5%		0	0.0%	8	5.4%		40	6.0%
Nationality-UN		0	0.0%		1	20.0%	65	13.5%		0	0.0%	14	9.5%		80	12.0%
Total		31	100.0%		5	100.0%	481	100.0%		3	100.0%	147	100.0%		667	100.0%

*p-value was calculated with the chi-square test by comparing the distribution in the other lineages combined. OR; odds ratio, CI; confidence interval, UN; unknown. Percentages are the ratios of the number of isolates belonging to each lineage.

As shown in Table 2, of the 321 isolates from patients aged 20–39 years, 219 (68.2%) were identified as lineage 2/Beijing. The number of lineage 2/Beijing isolates from Japanese patients was significantly higher than the number of isolates of other lineages from Japanese patients (odds ratio = 5.53, 95% confidence interval = 2.65–11.54, *p* < 0.01). Of the 481 isolates identified as lineage 2/Beijing, only 12 (2.5%) were from foreign-born patients (six from China and one each from the Philippines, Korea, Mongolia, Nepal, Indonesia, and other countries). Of the 147 isolates identified as lineage 4, only 8 (5.4%) were from foreign-born patients (including 3 from Brazil, 2 from Indonesia, 2 from Peru, and 1 from China). Isolates identified as lineages 2/Beijing and 4 were mainly from Japanese patients (84% and 85%, respectively).

### Phylogenetic analysis of lineage 1 isolates from the RYOKEN 2007 dataset  of the GReAT database

**Figure 1:**
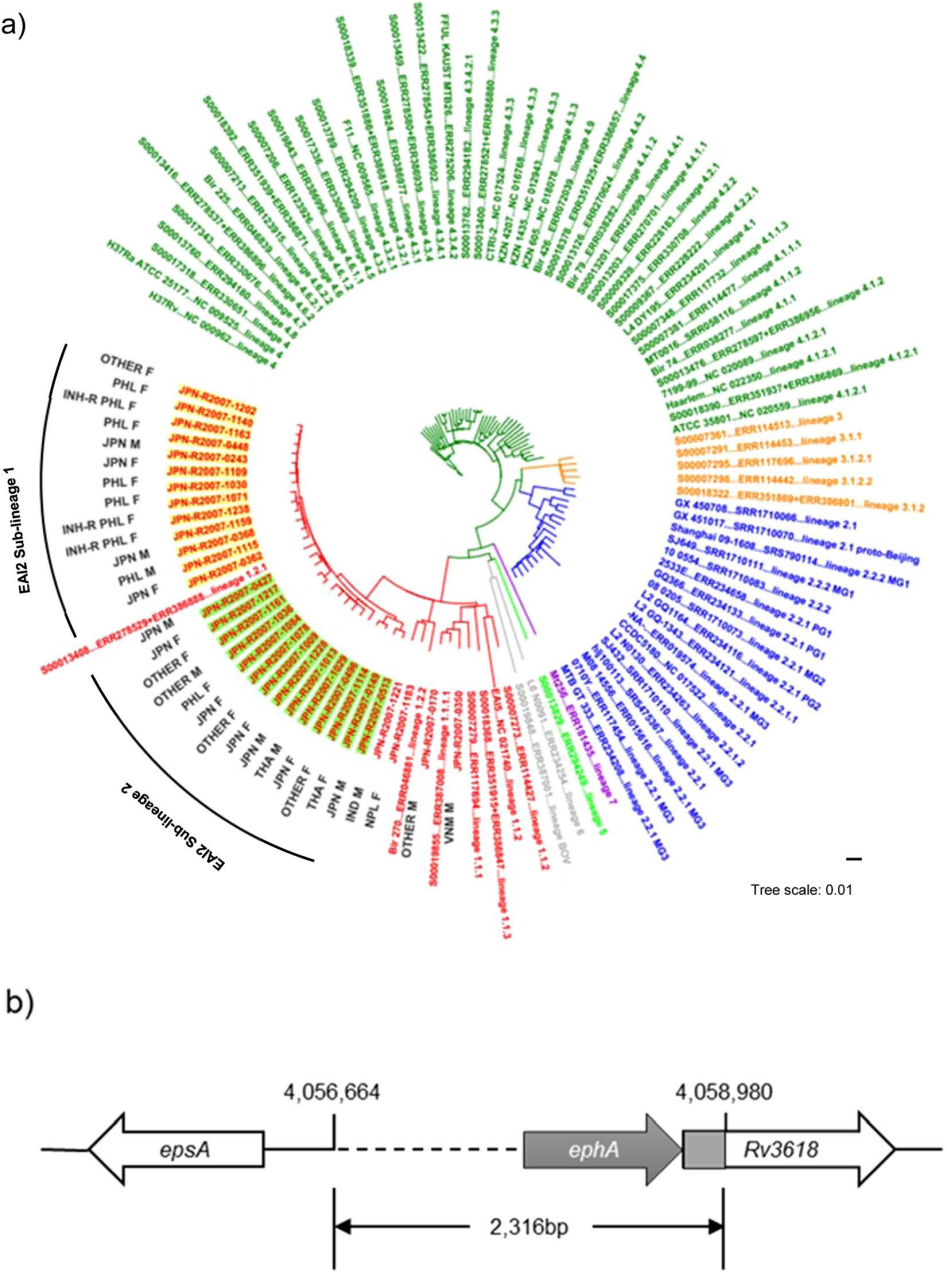
Phylogenetic analysis of the isolates belonging to lineage 1 in the RYOKEN 2007 dataset of the GReAT database. In total, 31 isolates from the RYOKEN 2007 dataset were classified into lineage 1 by TGS-TB. (**a**) Phylogenetic analysis of the isolates belonging to lineage 1. A phylogenetic tree was created based on SNV analysis of the core genome among the lineage 1 isolates with TGS-TB. Other strains were representative of each sub-lineage of lineage 1. M, male; F, female; IND, India; JPN, Japan; NPL, Nepal; PHL, the Philippines; THA, Thailand; VNM, Vietnam; OTHER, other foreign country; and INH-R, isoniazid resistant. Hatched rectangles are the sub-lineages of lineage 1.2.1 (EAI 2). (**b**) Schematic depiction of a large-scale deletion of 2,316 bp (4,056,664–4,058,980) observed in all isolates belonging to lineage 1.2.1 (EAI2) sub-lineage 1. The hatched line, grey arrow, and rectangle indicate the deleted regions in the genomes. *ephA* encodes epoxide hydrolase A.

A phylogenetic tree of 31 lineage 1 isolates from the RYOKEN 2007 dataset of the GReAT database generated by SNV-based analysis of the core genome with TGS-TB is shown in Fig. 1. No identical or closely related isolates were observed (Fig. 1 and Supplemental Table 1). The lineage 1 isolates were classified into three sub-lineage types. Of the 31 isolates, 27 (87.1%) were identified as lineage 1.2.1 [East-African-Indian (EAI)], two as lineage 1.2.2 (EAI1), and two as lineage 1.1.1.1 (EAI4). The isolates identified as lineage 1.2.2 (EAI1) were from foreign-born patients (Indonesia and Nepal). The isolates belonging to lineage 1.1.1.1 (EAI4) were also from foreign-born patients, one of whom was born in Vietnam. The isolates belonging to lineage 1.2.1 (EIA2) formed two clusters, namely, EAI2 sub-lineage 1 and EAI2 sub-lineage 2. Of the 13 EAI2 sub-lineage 1 isolates, 9 (69.2%) were from foreign-born patients, with most (8/9, 88.9%) from female patients from the Philippines, including three that were resistant to INH (Fig. 1). Furthermore, a 2,316-bp deletion (NC_000962.3, 4056664–4058980) was detected in the genomes of all isolates of sub-cluster EIA2 sub-lineage 1 (Supplemental Fig. 2). Of the 14 EAI2 sub-lineage 2 isolates, 7 (50%) were from foreign-born patients (Thailand or the Philippines) (Fig. 1). Among these EAI2 sub-lineages, there was no significant difference in the results of spoligotyping, which analysed the spacer regions of direct repeats in the genome of Mtb (Supplemental Table 2). A large deletion was observed in the EAI2 sub-lineage 1, which was the largest sub-lineage from the Philippines (data not shown). Taken together, the results show that this large deletion in sub-lineage 2 could be useful for identification of sub-lineage 1.2.1 (EAI2).

### Prediction of drug susceptibility by genomic analysis

The drug susceptibility of the isolates was predicted by TGS-TB, and the results were consistent with the mutations identified by TGS-TB^12^ and TB Profiler^14^. According to the genotyping results, 72 (10.8%) of the 667 isolates were resistant to at least one of the anti-TB drugs (Supplemental Table 4), 34 were resistant to INH, 9 to RFP, 40 to SM, 6 to EB, and 5 to LVFX (Table 3). In phenotypic method, 72 (10.8%) of the 667 isolates were resistant to at least one of the anti-TB drugs (Supplemental Table 4), 24 were resistant to INH, 2 to RFP, 48 to SM, 12 to EB, and 5 to LVFX (Table 3). The concordance ratio of INH, RFP, SM, EB, and LVFX between the genotypic and phenotypic methods was high, with values of 96.4%, 99.0%, 98.5%, 99.1%, and 100.0%, respectively, as were the specificities, with values of 97.4%, 98.9%, 99.9%, 100.0%, and 100.0%, respectively. However, the sensitivities to INH, RFP, SM, EB, and LVFX were 70.8%, 100.0%, 81.3%, 50.0%, and 100.0%, respectively. In particular, the sensitivities to INH, SM, and EB were low; therefore, unknown mutations in the genes associated with resistance to INH, SM, and EB were predicted by genomic analysis using TGS-TB ver. 2 (https://gph.niid.go.jp/tgs-tb/). The results identified mutations in candidate genes: *katG* (V230L, A256G, D259A, W397G, R463L L472R, P533S), *ndh* (L239V), *inhA* promoter (−40, −76), *gid* (G34A, D67H, L74S, G76D, V88G, E92D, L142F, S149R), D67H), and *embB* (G991S) (Supplemental Table 3). The genomic analysis results predicted low sensitivity of Mtb isolates to drugs (Table 3).

**Table 3:** Phenotypic DST, RYOKEN 2007, Japan.

Genotype^*^	Phenotype^†^	INH	RFP	SM	EB	LVFX
R	R	17	2	39	6	5
R	S	17	7	1	0	0
S	R	7	0	9	6	0
S	S	626	658	618	655	261
Concordance		96.4	99.0	98.5	99.1	100.0
Sensitivity		70.8	100.0	81.3	50.0	100.0
Specificity		97.4	98.9	99.8	100.0	100.0

^*^Genotypic DR of the isolates was predicted by TGS-TB, TB Profiler and KvarQ in TGS-TB. ^†^The methods of phenotypic DST of the isolates against isoniazid (INH), rifampicin (RFP), streptomycin (SM), ethambutol (EB), and levofloxacin (LVFX). R; resistant, S; susceptible. The DST method is described in the Materials and Methods section. The numbers of isolates is indicated in each column for anti-TB drugs. The concordance rate, sensitivity, and specificity of the genotypic method vs. phenotypic method are also indicated. The concordance, specificity, and sensitivity of prediction of drug susceptibility by TGS-TB were calculated by comparing the results of phenotypic DST, which was used as the gold standard.

## Discussion

This study was the first to identify the genetic characteristics of Mtb throughout Japan by WGS. The genomic analysis showed that the largest group of isolates belonged to lineage 2/Beijing, accounting for 485 (72.1%) of the 667 isolates analysed, whereas lineage 4 accounted for 147 (22.0%), and lineage 1 accounted for 31 (4.6%). Epidemiological analysis by WGS of foreign-born TB patients in countries with low TB prevalence has been conducted in the U.S.^21^, Canada^22^, Spain^23^, Italy^24^, and Germany^25^. Although most of these studies found limited evidence of the transmission of TB between foreign-born and native-born patients, the transmission of TB between foreigners and Japanese patients was not observed in the present study. Consistent with the increasing numbers of immigrants from Asian countries with high TB burdens, such as the Philippines, China, Vietnam, Nepal, and Indonesia, to Japan, the number of TB cases among foreign-born patients has also been increasing, going from 842 (3.3%) of 25,311 cases in 2007 to 1,338 (7.6%) of 17,625 cases in 2016. Of the 1,338 foreign-born TB patients in Japan identified in 2016, 318 (23.8%), 272 (20.3%), 212 (15.8%), 135 (10.1%), and 90 (6.7%) were from the Philippines, China, Vietnam, Nepal, and Indonesia, respectively (Tuberculosis in Japan: Annual Report 2017, http://www.jata.or.jp/rit/ekigaku/en/statistics-of-tb/). Therefore, to control TB from foreign-born patients, genetic markers from patients from each of these countries should be identified to predict the invasion and transmutation of TB.

As shown in Table 2, of the 31 isolates identified as lineage 1, 20 (64.5%) were from foreign-born TB patients (Table 2). The proportion of lineage 1 isolates from foreign-born TB patients in the Philippines was quite high compared with that of other lineages (0% for lineage 2, 2.5% for lineage 2/Beijing, 0% for lineage 3, and 5.4% for lineage 4). Kobayashi *et al*. reported that in Tokyo, the proportion of lineage 1 isolates was significantly higher in foreign-born patients than in Japanese patients^26^. Therefore, a greater prevalence of lineage 1 isolates would be expected from foreign-born patients. There were significant differences in the prevalence of lineage 1 isolates among groups, with foreign-born females aged 20–39 years being the most frequently infected. In particular, 14 isolates identified as lineage 1 (EAI2) from females from the Philippines aged 20–39 years formed a sub-cluster. These isolates were not closely related, as there were 86 to 289 SNVs among them (Supplemental Table 1). However, all 14 of these isolates had a large deletion of approximately 2.3 kb at the same position in the genome (Supplemental Fig. 2). The distribution of TB lineages in the present study was similar to that described in other reports using different methods, such as spoligotyping^20,27^. However, in the present study, spoligotyping was not able to distinguish the EA12 sub-lineage (Supplemental Table 2). The large deletion in sub-lineage 1 was also observed in a majority of the isolates from the Philippines in the GReAT database (unpublished data). Therefore, this deletion might be a heritable characteristic of sub-lineage 1 and could thus serve as a candidate genetic marker of the isolates originating from the Philippines.

The proportion of DR to at least one anti-TB drug was 9.6% in 2007 in Japan^11^, comparable to the value of 10.8% observed in the present study (Supplemental Table 4). The proposed method seems promising as a standard method for DST in the future. Indeed, the concordance and specificity of the genotyping method for predicting drug susceptibility was good compared with the method currently used (Table 3). However, the sensitivity differed among drugs (70.8%, 81.3%, 50.0%, and 100% for INH, SM, EB, and RFP, respectively) (Table 3).

The GReAT database and bacterial culture-based methods can also improve the prediction of DR in Mtb by detecting genomic mutations responsible for DR and reducing the time necessary for DR detection, from weeks and months to within days or hours^28^. To detect the mutation of the *rpoB* gene responsible for RFP resistance, gene targeting methods, such as line probe assays or the Cepheid Xpert Mtb/RIF assay, can provide DST results within days or hours. However, these methods can detect the most frequent mutations of the *rpoB* gene but are limited to RFP. With future improvements in sequencing methods, mutations associated with resistance to other anti-TB drugs can be obtained simultaneously by WGS. Bioinformatic tools, such as KvarQ^15^, TB Profiler^14^, PhyResSE^29^, CASTB^30^, Mykrobe Predictor^31^, and TGS-TB^12^, are also useful to predict DR to not only first-line but also second-line anti-TB drugs. However, predictions made with these tools are limited to the reported major mutations in the DR genes. Therefore, other relevant mutations in these genes may be unreported. In the present study, unreported mutations in the *katG*, *gid*, and *embB* genes responsible for resistance to INH and EB were identified (Supplemental Table 3). By updating the list of mutations in DR genes, these findings should contribute to the improvement of the prediction of DR in TB.

## Conclusion

WGS of 667 isolates from the RYOKEN 2007 dataset of the GReAT database revealed genomic markers of Mtb isolates from the Philippines (Fig. 1) and previously unknown mutations in genes associated with resistance to INH, SM, and EB (Supplemental Table 3) to improve genomics-based DST. This is the first report of WGS for anti-TB DST in Japan, showing the usefulness of the GReAT database.

## Supplementary information

Supplemental figure 1, Supplemental figure 2, Supplemental table 1, Supplemental table 2, Supplemental table 3, Supplemental table 4

## Data Availability

Data supporting the findings of this manuscript are available from the corresponding author upon reasonable request. Nucleotide sequence data reported are available in the DDBJ Sequenced Read Archive under the accession numbers DRA008824, DRA008825 and DRA008826.

## References

[1] Zammarchi L, Bartalesi F, Bartoloni A (2014). Tuberculosis in tropical areas and immigrants. Mediterranean journal of hematology and infectious diseases..

[2] He, X. C., Tao, N. N., Liu, Y., Zhang, X. X. & Li, H. C. Epidemiological trends and outcomes of extensively drug-resistant tuberculosis in Shandong, China. *BMC infectious diseases*. **17**(1), 555 (2017 Aug 9). 10.1186/s12879-017-2652-x PMC5551028 28793873

[3] Isaakidis P (2014). Alarming levels of drug-resistant tuberculosis in HIV-infected patients in metropolitan Mumbai, India. PloS one..

[4] Reechaipichitkul, W., Tubtim, S. & Chaimanee, P. Drug susceptibility patterns of Mycobacterium tuberculosis and clinical outcomes of drug-resistant tuberculosis at Srinagarind Hospital, a tertiary care center in northeastern Thailand. *The Southeast Asian journal of tropical medicine and public health*. **42**(5), 1154–62 (2011 Sep). 22299441

[5] Elmi Omar Sald, Hasan Habsah, Abdullah Sarimah, Mat Jeab Mat Zuki, Bin Alwi Zilfalil, Naing Nyi Nyi (2015). Multidrug-resistant tuberculosis and risk factors associated with its development: a retrospective study. The Journal of Infection in Developing Countries.

[6] Aye, S. *et al*. Evaluation of a tuberculosis active case finding project in peri-urban areas, Myanmar: 2014–2016. *International journal of infectious diseases: IJID: official publication of the International Society for Infectious Diseases* (2018 Feb 21). 10.1016/j.ijid.2018.02.012 29476901

[7] Walker T.M., Merker M., Kohl T.A., Crook D.W., Niemann S., Peto T.E.A. (2017). Whole genome sequencing for M/XDR tuberculosis surveillance and for resistance testing. Clinical Microbiology and Infection.

[8] Hasnain, S. E., O’Toole, R. F., Grover, S. & Ehtesham, N. Z. Whole genome sequencing: a new paradigm in the surveillance and control of human tuberculosis. *Tuberculosis* (*Edinb*). **95**(2), 91–4 (2015 Mar). 10.1016/j.tube.2014.12.007 25586521

[9] Nikolayevskyy V, Kranzer K, Niemann S, Drobniewski F (2016). Whole genome sequencing of Mycobacterium tuberculosis for detection of recent transmission and tracing outbreaks: A systematic review. Tuberculosis (Edinb)..

[10] Starks, A. M. *et al*. Collaborative Effort for a Centralized Worldwide Tuberculosis Relational Sequencing Data Platform. *Clinical infectious diseases: an official publication of the Infectious Diseases Society of America*. **61(**Suppl 3), S141–6 (2015 Oct 15). 10.1093/cid/civ610 PMC4583571 26409275

[11] Tuberculosis Research Committee (RYOKEN), Tokyo, Japan. Nationwide survey of anti-tuberculosis drug resistance in Japan. *Int J Tuberc Lung Dis*. **19**(2), 157–62 (2015 Feb). 10.5588/ijtld.13.0905 25574913

[12] Sekizuka T (2015). TGS-TB: Total Genotyping Solution for Mycobacterium tuberculosis Using Short-Read Whole-Genome Sequencing. PloS one..

[13] Organization WH. Laboratory services in tuberculosis control. Part III: culture. World Health Organization, Geneva, Switzerland (1998).

[14] Coll F (2015). Rapid determination of anti-tuberculosis drug resistance from whole-genome sequences. Genome medicine..

[15] Steiner A, Stucki D, Coscolla M, Borrell S, Gagneux S (2014). KvarQ: targeted and direct variant calling from fastq reads of bacterial genomes. BMC genomics..

[16] Jiang H, Lei R, Ding SW, Zhu S (2014). Skewer: a fast and accurate adapter trimmer for next-generation sequencing paired-end reads. BMC bioinformatics..

[17] Li, H & Durbin, R. Fast and accurate long-read alignment with Burrows-Wheeler transform. *Bioinformatics*. **26**(5), 589–95 (2010 Mar 1). 10.1093/bioinformatics/btp698 PMC2828108 20080505

[18] Cole, S. T. *et al*. Deciphering the biology of Mycobacterium tuberculosis from the complete genome sequence. *Nature*. **393**(6685), 537–44 (1998 Jun 11). 10.1038/31159 9634230

[19] Stamatakis Alexandros (2014). RAxML version 8: a tool for phylogenetic analysis and post-analysis of large phylogenies. Bioinformatics.

[20] Kamerbeek, J. *et al*. Simultaneous detection and strain differentiation of Mycobacterium tuberculosis for diagnosis and epidemiology. *Journal of clinical microbiology*. **35**(4), 907–14 (1997 Apr). 10.1128/jcm.35.4.907-914.1997 PMC229700 9157152

[21] Salinas Jorge L., Mindra Godwin, Haddad Maryam B., Pratt Robert, Price Sandy F., Langer Adam J. (2016). Leveling of Tuberculosis Incidence — United States, 2013–2015. MMWR. Morbidity and Mortality Weekly Report.

[22] Asadi, L., Heffernan, C., Menzies, D. & Long, R. Effectiveness of Canada’s tuberculosis surveillance strategy in identifying immigrants at risk of developing and transmitting tuberculosis: a population-based retrospective cohort study. *The Lancet Public health*. **2**(10), e450–e7 (2017 Oct). 10.1016/S2468-2667(17)30161-5 29253429

[23] Borrell S., Tudó G., Rey E., González-Martín J., Español M., March F., Coll P., Orcau A., Caylà J.A., Jansà J.M., Alcaide F., Martín-Casabona N., Salvadó M., Martinez J.A., Vidal R., Sanchez F., Altet N. (2010). Tuberculosis transmission patterns among Spanish-born and foreign-born populations in the city of Barcelona. Clinical Microbiology and Infection.

[24] Ingrosso L (2014). Risk factors for tuberculosis in foreign-born people (FBP) in Italy: a systematic review and meta-analysis. PloS one..

[25] Diel, R., Rusch-Gerdes, S. & Niemann, S. Molecular epidemiology of tuberculosis among immigrants in Hamburg, Germany. *Journal of clinical microbiology*. **42**(7), 2952–60 (2004 Jul). 10.1128/JCM.42.7.2952-2960.2004 PMC446315 15243044

[26] Kato-Miyazawa M., Miyoshi-Akiyama T., Kanno Y., Takasaki J., Kirikae T., Kobayashi N. (2015). Genetic diversity of Mycobacterium tuberculosis isolates from foreign-born and Japan-born residents in Tokyo. Clinical Microbiology and Infection.

[27] Montoya, J. C., Murase, Y., Ang, C., Solon, J. & Ohkado, A. A molecular epidemiologic analysis of Mycobacterium tuberculosis among Filipino patients in a suburban community in the Philippines. *Kekkaku: [Tuberculosis]*. **88**(6), 543–52 (2013 Jun). 23898494

[28] Moore, D. A. & Shah, N. S. Alternative methods of diagnosing drug resistance–what can they do for me? *The Journal of infectious diseases*. **204**(Suppl 4), S1110–9 (2011 Nov 15). 10.1093/infdis/jir448 PMC3192546 21996693

[29] Feuerriegel Silke, Schleusener Viola, Beckert Patrick, Kohl Thomas A., Miotto Paolo, Cirillo Daniela M., Cabibbe Andrea M., Niemann Stefan, Fellenberg Kurt (2015). PhyResSE: a Web Tool Delineating Mycobacterium tuberculosis Antibiotic Resistance and Lineage from Whole-Genome Sequencing Data. Journal of Clinical Microbiology.

[30] Iwai, H., Kato-Miyazawa, M., Kirikae, T. & Miyoshi-Akiyama, T. CASTB (the comprehensive analysis server for the Mycobacterium tuberculosis complex): A publicly accessible web server for epidemiological analyses, drug-resistance prediction and phylogenetic comparison of clinical isolates. *Tuberculosis* (*Edinb*). **95**(6), 843–4 (2015 Dec). 10.1016/j.tube.2015.09.002 26542225

[31] Bradley P (2015). Rapid antibiotic-resistance predictions from genome sequence data for Staphylococcus aureus and Mycobacterium tuberculosis. Nature communications..

