# Supplementary material for: Whole-genome sequencing-based epidemiological analysis of anti-tuberculosis drug resistance genes in Japan in 2007: Application of the Genome Research for Asian Tuberculosis (GReAT) database
Source: Sci Rep. 2019 Sep 6;9:12823. doi: 10.1038/s41598-019-49219-5 (PMC6731343; doi:10.1038/s41598-019-49219-5)
Supplement: Supplementary file 1 — Supplemental figure 1, Supplemental figure 2, Supplemental table 1, Supplemental table 2, Supplemental table 3, Supplemental table 4 [file 41598_2019_49219_MOESM1_ESM.docx]

**Whole-genome sequencing-based epidemiological analysis of anti-tuberculosis drug resistance genes in Japan in 2007: Application of the Genome Research for Asian Tuberculosis (GReAT) database**

Takemasa Takii

Kouhei Seki

Yasutaka Wakabayashi

Yuta Morishige

Tsuyoshi Sekizuka

Akifumi Yamashita

Kengo Kato

Kazuhiro Uchimura

Akihiro Ohkado

Naoto Keicho

Satoshi Mitarai

Makoto Kuroda

Seiya Kato


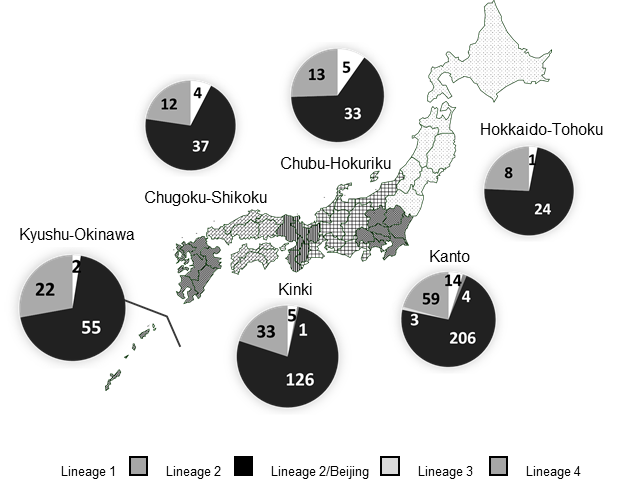


**Supplemental figure 1. Distribution of tuberculosis patients belonging to each lineage in the Japanese geographical region.**

Six hundred and sixty-seven isolates were from tuberculosis patients in Japan (RYOKEN 2007) and were classified into each lineage by TGS-TB: lineage 1, white; 2, hatched line; 2/Beijing, black; 3, grid; and 4, light grey. The six typical classifications of the Japanese geographical region, namely, Hokkaido-Tohoku, Kanto, Chubu-Hokuriku, Kinki, Chugoku-Shikoku, and Kyushu-Okinawa, are indicated. The numbers in each graph are the number of patients in the region.


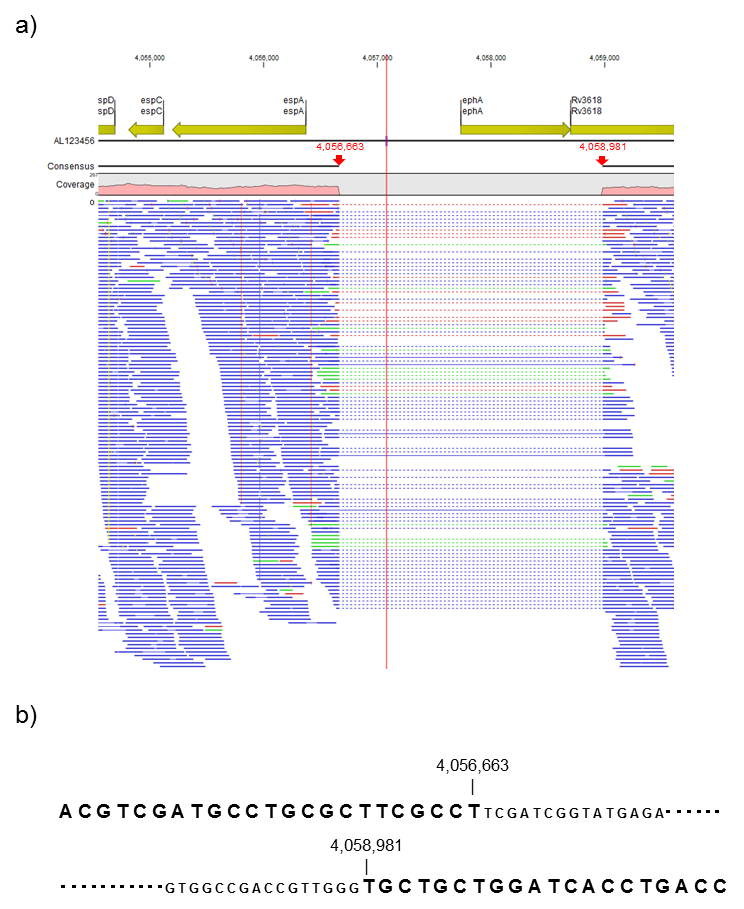


**Supplemental figure 2. Lineage 1.2.1, EAI2, sub-lineage 1-specific deletion in the genome of JPN-R2007-1202.**

a) Large-gap read mapping analysis of JPN-R2007-1202, the representative EAI2, sub-lineage 1 isolate, using CLC Genomics Workbench (World Fusion, CLC bio Japan, Inc., Kobe, Japan). The red coloured arrows on the panel indicate the start and end positions of the large deletion of 2,316 bp. b) The sequence around the deletion site. Small letters indicate deletions from the gene sequence. The numbers 4,056,664 and 4,058,980 indicate the position in the reference genome NC_000962.3.

| **Supplemental Table 2. *in silico* spoligotyping of the lineage 1 isolates.** | | | |  |
| --- | --- | --- | --- | --- |
| Strain ID | spoligotype (octal) ^*^ | SIT^†^ | Clade^†^ | EAI2 sub-lineage^‡^ |
| JPN-R2007-0149 | - | - | - | sub-lineage 2 |
| JPN-R2007-0170 | 777777774413771 | 139 | EAI4-VNM | - |
| JPN-R2007-0243 | 677777477413771 | 19 | EAI2-Manilla | sub-lineage 1 |
| JPN-R2007-0350 | 777737777413771 | 618 | EIA5 | - |
| JPN-R2007-0362 | 677777477413771 | 19 | EAI2-Manilla | sub-lineage 1 |
| JPN-R2007-0368 | 677777477413771 | 19 | EAI2-Manilla | sub-lineage 1 |
| JPN-R2007-0427 | 677777477413771 | 19 | EAI2-Manilla | sub-lineage 2 |
| JPN-R2007-0448 | 677777477413771 | 19 | EAI2-Manilla | sub-lineage 1 |
| JPN-R2007-0496 | 677777477413771 | 19 | EAI2-Manilla | sub-lineage 2 |
| JPN-R2007-0513 | 677777477413771 | 19 | EAI2-Manilla | sub-lineage 2 |
| JPN-R2007-1009 | - | - | - | sub-lineage 2 |
| JPN-R2007-1013 | - | - | - | sub-lineage 2 |
| JPN-R2007-1029 | 674000003413771 | 89 | EAI2-nonthaburi | sub-lineage 2 |
| JPN-R2007-1030 | 677777477413771 | 19 | EAI2-Manilla | sub-lineage 1 |
| JPN-R2007-1036 | 677777477413771 | 19 | EAI2-Manilla | sub-lineage 2 |
| JPN-R2007-1071 | 677777477413771 | 19 | EAI2-Manilla | sub-lineage 1 |
| JPN-R2007-1078 | 677777477413771 | 19 | EAI2-Manilla | sub-lineage 2 |
| JPN-R2007-1084 | 677777477413771 | 19 | EAI2-Manilla | sub-lineage 2 |
| JPN-R2007-1109 | 677777477413771 | 19 | EAI2-Manilla | sub-lineage 1 |
| JPN-R2007-1114 | 677777477413771 | 19 | EAI2-Manilla | sub-lineage 2 |
| JPN-R2007-1115 | 677777477413771 | 19 | EAI2-Manilla | sub-lineage 1 |
| JPN-R2007-1140 | 677777477413771 | 19 | EAI2-Manilla | sub-lineage 1 |
| JPN-R2007-1159 | 677777477413700 | 758 | EAI2-Manilla | sub-lineage 1 |
| JPN-R2007-1161 | 677777477413771 | 19 | EAI2-Manilla | sub-lineage 2 |
| JPN-R2007-1163 | - | - | - | sub-lineage 1 |
| JPN-R2007-1183 | 777777777413700 | 138 | EAI5 | - |
| JPN-R2007-1202 | 677777477413771 | 19 | EAI2-Manilla | sub-lineage 1 |
| JPN-R2007-1217 | 677777477413771 | 19 | EAI2-Manilla | sub-lineage 2 |
| JPN-R2007-1221 | 774777777413731 | 1435 | EAI-SOM | - |
| JPN-R2007-1228 | 677777477413771 | 19 | EAI2-Manilla | sub-lineage 2 |
| JPN-R2007-1238 | 677777477413771 | 19 | EAI2-Manilla | sub-lineage 1 |
| ^*^The results of in silico spoligotyping were analyzed by TGS-TB (ref). ^†^SIT and clade of each isolate were analyzed by SITVIT WEB (http://www.pasteur-guadeloupe.fr:8081/SITVIT_ONLINE/). ^‡^The sub-lineages of EAI2 of the isolates were classified in this study. -; un-identified or inapplicable. | | | | |
|  |  |  |  |  |

| **Supplemental Table 4. Numbers of drug resistant Mtb in genomic and phenotypic methods.** | | | | | | |
| --- | --- | --- | --- | --- | --- | --- |
|  | Number of the resistant drugs | | DR prediction based on WGS |  | DST |  |
|  | 1 |  | 58 |  | 58 |  |
|  | 2 |  | 12 |  | 8 |  |
|  | 3 |  | 2 |  | 6 |  |
|  | 4 |  | 0 |  | 0 |  |
|  | 5 |  | 0 |  | 0 |  |
|  | Total |  | 72 |  | 72 |  |
